# Dr. J. Falk

**Published:** 1889-06

**Authors:** 


DR. J. FALK, whose practice is limited to diseases of the eye and ear, has removed his office to No. 421 Pearl street.

